# Combining Network Pharmacology and Transcriptomic Strategies to Explore the Pharmacological Mechanism of Total Ginsenoside Ginseng Root and Its Impact on Antidepressant Effects

**DOI:** 10.3390/ijms252312606

**Published:** 2024-11-24

**Authors:** Weijia Chen, Pengli Guo, Lili Su, Xiangjuan Guo, Meiling Shi, Jianan Geng, Ying Zong, Yan Zhao, Rui Du, Zhongmei He

**Affiliations:** 1 College of Chinese Medicinal Materials, Jilin Agricultural University, Changchun 130118, China; weijiac@jlau.edu.cn (W.C.); gengjianan@jlau.edu.cn (J.G.); zongying7699@126.com (Y.Z.); zhaoyan@jlau.edu.cn (Y.Z.); 2 Jilin Provincial Engineering Research Center for Efficient Breeding and Product Development of Sika Deer, Changchun 130118, China; 3 Key Laboratory of Animal Production and Product Quality and Security, Ministry of Education, Ministry of National Education, Changchun 130118, China

**Keywords:** depression, TGGR, network pharmacology, transcriptome sequencing, AMPK-SIRT1-PGC-1α pathway

## Abstract

Depression is one of the most common neurological diseases, which imposes a substantial social and economic burden on modern society. The purpose of this study was to explore the mechanism of total ginsenoside ginseng root (TGGR) in the treatment of depression through a comprehensive strategy combining network pharmacology, transcriptomics, and in vivo experimental validation. The Traditional Chinese Medicine Systematic Pharmacology (TCMSP) database and literature were used to collect the main components and targets of TGGR. Gene Ontology (GO) and Kyoto Encyclopedia of Genes and Genomes (KEGG) analyses were applied to explore the underlying mechanisms. In addition, the chronic unpredictable mild stress (CUMS)-induced C57BL/6 mouse model was used to evaluate the antidepressant activity of TGGR. The results showed that TGGR improved depression-like behavior in mice and increased the decrease in serum 5-hydroxytryptamine (5-HT) and brain-derived neurotrophic factor (BDNF) levels caused by CUMS. Combined network pharmacology and transcriptomic analysis showed that the AMP-activated kinase (AMPK) signaling pathway mainly enriched the core target. Immunohistochemistry, Western blotting, and reverse transcription quantitative polymerase chain reaction (RT-qPCR) were used to confirm whether TGGR exerts antidepressant effects by regulating this pathway. The results showed that TGGR has a regulatory impact on related proteins in the AMPK pathway, and the regulatory effect of TGGR on proteins was inhibited after the administration of related pathway inhibitors. In summary, total ginsenosides may regulate the AMPK signaling pathway and activate the sirtuin 1 (SIRT1) peroxisome proliferator-activated receptor-gamma coactivator 1-alpha (PGC-1α) pathway to have therapeutic effects on depression.

## 1. Introduction

Depression is a mood disorder that places a massive burden on society and individuals [1], and is characterized by persistent depressed mood, decreased interest, cognitive impairment, sleep disturbances, and suicidal tendencies, which severely reduce the psychosocial function and quality of life of patients [2]. It is expected to be one of the top three causes of disease burden by 2030 [3]. The pathogenesis of depression is very complex, involving many factors, such as genetic, physiological, social, and psychological factors. There are many hypotheses about the pathogenesis of depression, including monoamine neurotransmitters, neurotrophic factors, inflammation, neuroplasticity, and the hypothalamic–pituitary–adrenal axis, among which the monoamine neurotransmitter hypothesis is the most concerning one at present [4]. Although there have been many hypotheses about the pathogenesis of depression, its specific pathogenesis and causes are still unclear. Therefore, this study combined transcriptomics and network pharmacology techniques to explore whether TGGR can treat depression through predicted targets and pathways and to explore its specific mechanism of action. Some drugs based on monoamine neurotransmitters have been used to reduce depressive symptoms. These drugs have particular efficacy but also shortcomings, such as significant side effects, single targets, severe adverse reactions, and lagging efficacy [5]. Therefore, the search for new drugs to alleviate or treat depression has significant clinical and social value.

*Panax ginseng C. A. Mey*, a perennial herb of the Acantharean family, is one of the most famous and valuable forms of traditional herbs and has been widely used for thousands of years. TGGR is the main active component of ginseng. Ginseng and TGGR have been shown to have multiple beneficial functions in the brain, including antidepressant or anti-stress effects [6]. In addition, TGGR also has abundant pharmacological activities, which not only include anti-inflammatory, anti-tumor, anti-fatigue, and immunity enhancement effects but also significant regulatory effects on the central nervous system, mainly manifested in enhancing the excitatory and inhibitory processes of the cerebral cortex [7]. Modern pharmacological studies have also found that ginseng can play a neuroprotective role through the blood–brain barrier and has a therapeutic effect on some neurodegenerative diseases, including depression [8,9,10]. Currently, about 250 ginsenosides have been isolated and identified, which are mainly divided into three groups: protopanaxadiols (PPDs, such as ginsenoside Rb1, Rb2, Rc, Rd, and Rg3); protopanaxatriols (PPTs, such as ginsenoside Re, Rf, Rg1, and Rh1), and oleanolic acids (OAs, such as ginsenoside Ro) [11]. Many ginsenoside monomers and crude extracts have been shown to have antidepressant effects. For example, ginseng extract G115, a standardized extract of ginseng, showed antidepressant effects in ethanol-treated mouse models by increasing levels of brain-derived neurotrophic factor (BDNF) in the hippocampus and prefrontal cortex [12]. Ginsenoside Rg1 increased connexin43 (Cx43) protein levels by upregulating Cx43 mRNA and downregulating Cx43 degradation to improve depression-like symptoms in mice [13]. Ginsenoside Rk3 alleviates depression-like behavior in mice by improving disorder of the hypothalamic–pituitary–adrenal axis, targeting tryptophan hydroxylase, and reshaping the intestinal microenvironment [14]. Due to their large numbers, ginsenoside Re, Rd, Rf, Rg3, Rc, Rg1, Rb1, and protopanaxadiol with higher content were screened for further experiments.

SIRT1 is a Nicotinamide Adenine Dinucleotide (NAD^+^)-dependent deacetylase with multiple functions in DNA repair; transcriptional recombination; and combating stress, oxidative stress, inflammation, and apoptosis [15,16,17]. So far, there have been many studies linking SIRT1 to depression. For example, in a clinical trial where SIRT1 was first identified as a gene associated with major depressive disorder (MDD), SIRT1 was significantly downregulated in blood samples from MDD patients compared to healthy people [18]. In animal models of depression, treatment with resveratrol, a well-known SIRT1 activator, improved the excessive anxiety state and reduced depression-like behavior in Wistar–Kyoto (WKY) rats [19]. In addition, SIRT1 plays an important role in regulating cellular metabolism and mitochondrial health by regulating the transcription of PGC-1α [20,21]. Activation of SIRT1 also promotes mitochondrial protein recovery and function by increasing mitochondrial biogenesis [22].

Network pharmacology can demonstrate the synergistic effect of combination drugs, traditional Chinese medicines, and drug monomers on multiple targets and pathways by constructing an interaction network of various compounds, targets, and pathways [23]. Nevertheless, network pharmacology is based on public databases and can only predict the potential outcome. Transcriptome sequencing analysis can identify and visualize changes in gene expression profiles during drug interference in disease development, demonstrate gene function and structure at the whole level, and reveal the molecular mechanisms underlying the drug’s role in disease [24,25]. The CUMS model is a widely used animal model of depression [26], and antidepressants can effectively reverse most of the depressive-like symptoms of CUMS. Thus, the integration of network pharmacology and transcriptomics helps to overcome the limitations of the lack of experimental basis for the former and the lack of molecular mechanism explanation in the latter, provides new targets and pathways for drugs to treat diseases, and provides new research strategies [27]. For this study, combining network pharmacology with transcriptomics would help to understand better the mechanism of antidepressant action of TGGR on CUMS -induced depression.

Therefore, based on the CUMS mouse model, this study first explored the potential targets and mechanisms of action of TGGR in the treatment of depression through transcriptomics combined with network pharmacology. Immunohistochemistry, transmission electron microscopy, Western blotting, and RT-qPCR validated the transcriptomic and network pharmacological results. These provide a theoretical basis for the scientific research and clinical application of TGGR.

## 2. Results

### 2.1. Network Pharmacological Analysis

**Figure 1:**
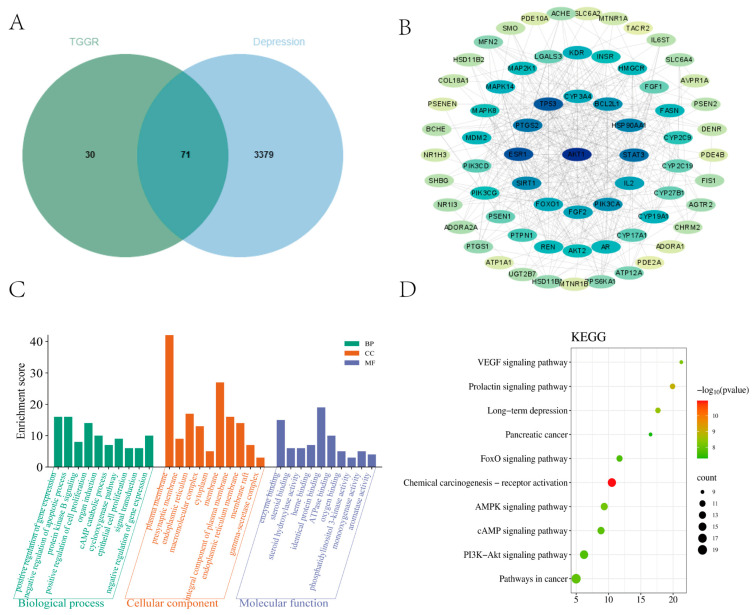
Potential targets of TGGR antidepressants and enrichment analyses. (**A**) Ginsenoside and depression target Venn plots. (**B**) Construction of the intersection target PPI network map of ginsenoside. (**C**,**D**) Gene Ontology and Kyoto Encyclopedia of Genes and Genomes enrichment analyses.

A total of 101 ginsenoside-related targets were obtained through the PharmMapper database, and 3450 depression-related targets were obtained from the DisGeNET database: Disease gene association database. The intersection of ginsenoside and depression targets yielded 71 intersection targets (see Figure 1A).

The 71 intersection genes were imported into the String database to build the Protein-protein interaction (PPI) interaction network (see Figure 1B). GO and KEGG enrichment analyses were performed for intersection genes. They were screened with *p* < 0.05, and the GO enrichment analysis yielded 313 functional relationships. Among them, 207 participated in biological processes (BP), mainly involving positive regulation of gene expression, negative regulation of the apoptotic process, protein kinase B signaling, positive regulation of cell proliferation, positive regulation of protein phosphorylation, and so on. The cell composition (CC) in 40 functional relationships mainly involved the phosphatidylinositol 3-kinase complex, cell cortex, perinuclear region of cytoplasm, class IB, and so on. Molecular functions (MF) in 66 functional relationships mainly involved enzyme binding, steroid binding, RNA polymerase II transcription factor activity, ligand-activated sequence-specific DNA binding, heme binding, identical protein binding, and so on. Several significant biological processes were analyzed in the online platform, and the results are shown in Figure 1C. Similarly, screening of KEGG pathways at *p* < 0.05 yielded 130 signaling pathways. KEGG enrichment showing ginsenoside antidepressant effect-related pathways included Chemical carcinogenesis—receptor activation, Prolactin signaling pathway, EGFR tyrosine kinase inhibitor resistance, Kaposi sarcoma-associated herpesvirus infection, and so on. Several more significant pathways were uploaded to the online platform for visual processing, and the results are shown in Figure 1D. Most importantly, the analysis results were also imported into the Cytoscape 3.9.1 software to construct the ginseng–ginsenoside–depression–pathway–target network map, to explore the relationship between eight active compounds and 71 overlapping targets (See Figure S1E in the Supplementary Material) and analyze the network topological parameters by using the plugin cytoNCA, The top 10 genes were selected based on the degree value (degree); these were: *AKT1* (degree = 39), *ESR1* (=34), *TP53* (=30), *STAT3* (=29), *PTGS2* (=27), *HSP90AA1* (=26), *SIRT1* (=25), *PIK3CA* (=25), *BCL2L*1 (=22), and *IL2* (=22).

### 2.2. Effect of TGGR on Depression-like Behavior Induced by CUMS in Mice

**Figure 2:**
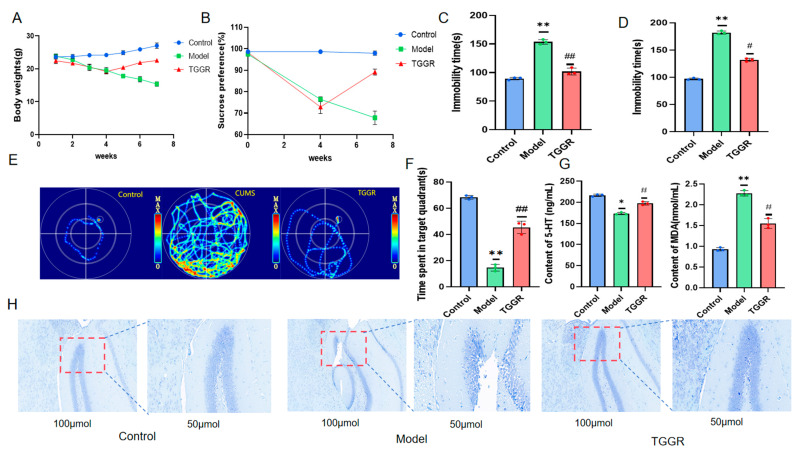
(**A**) Effects of TGGR on body weight of mice. (**B**) The effect of TGGR on sucrose preference in mice. (**C**,**D**) Effects of TGGR administration on resting time of FST and TST mice. (**E**) Heat map of MWM mouse activity. (**F**) The residence time of mice in the MWM target quadrant. (**G**) The levels of serum 5-HT and MDA, determined by ELISA. (**H**) Apoptosis of hippocampal neurons in each group (Nissl staining).The second picture for each group is a larger picture inside the red dashed box of the first picture. * *p* < 0.05 and ** *p* < 0.01 were significantly different from the control group, and ^#^ *p* < 0.05 and ^##^ *p* < 0.01 were significantly different from model group.

During the whole experiment, the weight of the mice in the control group increased steadily. Compared with the control group, the body weight of mice in the CUMS model group was significantly reduced. After TGGR was administered, the situation was improved considerably (Figure 2A). Secondly, the antidepressant effect of TGGR was evaluated by four behavioral manifestations: sucrose preference test (SPT), forced swimming Test (FST), tail suspension test (TST), and morris water maze (MWM).

As shown in Figure 2B–F, the sucrose preference score and the number of times that CUMS model mice were upright was significantly reduced compared with the control group. Similar results were found for TST and FST. The immobile time for FST and TST in CUMS mice was considerably longer than that in the control group, which indicated that the animals had anhedonia, decreased motor ability and exploration ability, and showed the core symptoms of depression. The MWM test also showed that model mice spent less time in the target quadrant compared to the control group. These behavioral changes can be improved after TGGR treatment. These findings suggest that TGGR improves depression-like behavior in mice induced by CUMS.

### 2.3. Effects of TGGR on CUMS-Induced Neuronal Apoptosis and Depression-Related Indexes in Mice

5-HT is an important neurological marker to identify depression in clinical studies [28]; MDA is one of the essential indicators to measure oxidative damage in the body and is often used to detect depression in clinical practice. Enzyme linked immunosorbent assay (ELISA) kit was used to detect 5-HT and MDA levels in plasma. As shown in Figure 2G, compared with the control group, the levels of 5-HT in CUMS model mice were significantly decreased, and the levels of MDA were significantly increased. After TGGR administration, the concentrations of 5-HT significantly increased, and the concentrations of MDA significantly decreased. The survival rate of hippocampal neurons was measured by Nissl staining. As shown in Figure 2H, compared with mice in the control group, a large number of neurons in the CUMS-treated group showed acidosis, nuclear shrinkage, and cell structure loss. On the contrary, TGGR can significantly reduce the degree of neuronal degeneration and necrosis induced by CUMS.

### 2.4. Effect of TGGR on Transcriptomics Profile in CUMS-Induced Mice

**Figure 3:**
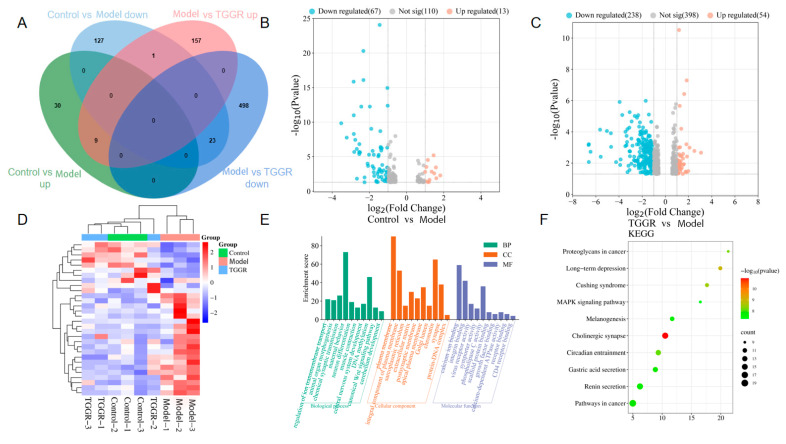
(**A**) DEGs crossover results in the control group, CUMS group, and TGGR group. (**B**,**C**) DEG volcanic distribution map. Blue dots indicate downregulated mRNA; red dots indicate upregulated mRNA. (**D**) Heat map analysis of low- and high-expression DEG. (**E**) GO enrichment analysis was performed on DEGs’ cellular components, biological processes, and molecular functions. The first 10 items are shown in the figure. (**F**) The top 10 pathways of DEGs after performing KEGG enrichment.

Transcriptomics techniques have recently been regarded as a reliable and precise method to search the differential expressed genes (DEGs) and predict potential targets and underlying mechanisms [29]. An RNA sequencing (RNA-seq) transcriptomics strategy was performed to decode the antidepression mechanism of TGGR and find DEGs in the hippocampus of CUMS-induced mice treated with or without TGGR. According to the results, compared with CUMS group, 190 DEGs were detected in the control group, among which 39 DEGs were significantly upregulated, and 151 DEGs were significantly downregulated. Similarly, 688 DEGs were detected in the TGGR group, among which 167 DEGs were significantly upregulated and 521 DEGs were significantly downregulated compared with CUMS group. Compared with the model group, 9 genes were significantly upregulated in the control group and dose group and 23 genes were significantly downregulated in the control group and administration group, indicating that 32 differential genes changed after CUMS modeling were adjusted after the application of TGGR, and that TGGR may achieve an antidepressant effect based on these genes, as shown in the Figure 3A. Figure 3B,C show the volcanic distribution of DEGs. Blue dots indicate mRNA downregulation, and red dots indicate mRNA upregulation. The data were analyzed to obtain the differential gene cluster diagram. It can be seen that there are more genes from the TGGR group clustered with the genes from the control group, as shown in Figure 3D, with the abscissa representing different samples.

Through GO enrichment analysis, we found that 232 GO entries could be enriched in 845 expressed DEGs regulated by TGGR when *p* < 0.05. There were 84 entries in the biological process, including the neuromuscular process, regulation of neuronal synaptic plasticity, etc. There were 24 entries of cellular components, mainly including centrosome, postsynaptic membrane, etc., and 124 molecular functional entries, such as regulation of neuronal synaptic plasticity, nitric oxide, and stimulation of guanylate cyclase. The GO enrichment analysis of the top 10 with high expression of DEGs, in which TGGR exerts antidepressant effects, is shown in Figure 3E. In addition, we performed a KEGG pathway enrichment analysis of TGGR-regulated DEGs. A total of 845 highly expressed DEGs could be enriched in 64 signaling pathways; the first 10 pathways are shown in Figure 3F, and mainly include long-term depression and the mitogen-activated protein kinase (MAPK) signaling pathway, etc.

### 2.5. Combining Network Pharmacology with Transcriptomics to Analyze the Key Targets and Pathways of TGGR in the Prevention and Treatment of Depression

**Figure 4:**
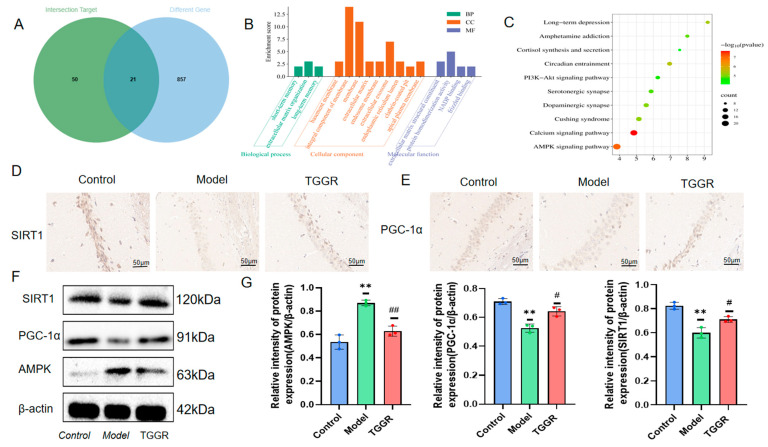
The key targets and pathways of TGGR in the prevention and treatment of depression and the preliminary validation of this target and pathway of action, analyzed with a combination of network pharmacology and transcriptomics. (**A**) Cross-targets of TGGR and depression in network pharmacology and cross-targets of transcriptomic differential genes. (**B**) Cross-target GO enrichment analysis. (**C**) The top 10 KEGG enrichment pathways for cross-targets. (**D**,**E**) Expression of SIRT1 and PGC-1α in mouse hippocampus (immunohistochemical staining). (**F**) Western blot analysis of AMPK, SIRT1, and PGC-1α in the hippocampus of mice in each group. Protein expression was normalized to β-actin for quantitative analysis, and its value expressed as an average (**G**). Normalization of the data to β-actin. Values are expressed as average ± SD. ** *p* < 0.01 was significantly different from the control group, and ^#^ *p* < 0.05 and ^##^ *p* < 0.01 were significantly different from model group.

The cross-targets of TGGR and depression in network pharmacology were intersected with differential genes in the transcriptome using the Weisengxin platform, and the key targets of TGGR and depression were obtained, as shown in Figure 4A. The key targets were imported into the DAVID platform for GO and KEGG enrichment analysis. A total of 21 key crossover targets were mainly enriched into 16 GO entries, as shown in Figure 4B. The results of the KEGG enrichment analysis primarily include the Phosphatidylinositol 3-kinase-protein kinase B (PI3K-Akt) signaling pathway, long-term depression, the AMPK signaling pathway, and other signaling pathways, as shown in Figure 4C. The study found that the path was highly consistent with the pathway that ranked high in network pharmacology and showed most of the above pathways in the transcriptome analysis results, indicating that the results of the two prediction methods were consistent. The results show that the combined analysis of network pharmacology and transcriptomics can help explore critical targets and pathways for treating TGGR antidepressants. Therefore, AMPK signaling pathways with the best enrichment and high coincidence with network pharmacology and transcriptomics were selected for subsequent validation.

### 2.6. Validation of the Target and Mechanism of TGGR in the Treatment of Depression

SIRT1 is the central downstream molecule of the AMPK signaling pathway. Studies have shown that AMPK is involved in energy metabolism and the development of anxiety and depression-like behaviors through SIRT1 [30,31,32]. In addition, SIRT1 often increases mitochondrial biosynthesis by activating deacetylated PGC-1α, so we selected SIRT1 as a key protein in subsequent mitochondrial energy metabolism. We first detected the expression of SIRT1 and PGC-1α in the hippocampus of mice by the immunohistochemical method. Then, protein expression levels of AMPK, SIRT1, and PGC-1α were detected by immunoblotting, to verify whether TGGR exerts antidepressant effects by modulating the AMPK signaling pathway. As shown in Figure 4D,E, the positive signal intensity of SIRT1 and PGC-1α in the hippocampus of Model group mice was significantly decreased, and the positive signal intensity of SIRT1 and PGC-1α was significantly increased after TGGR treatment. Western blot results in Figure 4F showed that CUMS stimulation increased AMPK levels and decreased SIRT1 and PGC-1α expression compared with controls. Compared with the model group, TGGR treatment reversed the effects of CUMS on AMPK, SIRT1, and PGC-1α. It could be seen that after TGGR administration, the expression levels of SIRT1 and PGC-1α were significantly increased, while the expression levels of AMPK were significantly decreased. Quantitative results are shown in Figure 4G. These results suggest that the antidepressant mechanism of TGGR may be related to the AMPK–SIRT1 signaling pathway.

### 2.7. Effect of TGGR on Depression-like Behavior in CUMS Model Mice in the Presence of the SIRT1-Specific Inhibitor Selisistat (EX-527)

**Figure 5:**
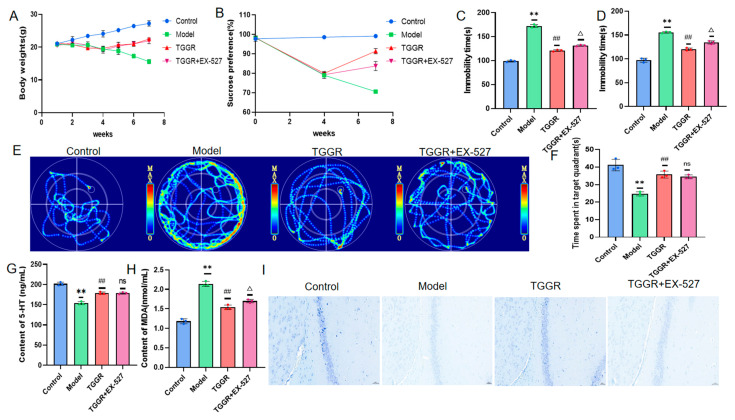
(**A**) Changes in body weight of mice. (**B**) Changes in sucrose preference of mice. (**C**,**D**) Effects of TGGR administration on resting time of TST and FST mice. (**E**) Heat maps of MWM mouse activity. (**F**) Residence time of mice in the MWM target quadrant. (**G**,**H**) ELISA-detected serum 5-HT and MDA levels. (**I**) Apoptosis of hippocampal neurons in each group (Nissl staining). ** *p* < 0.01 was significantly different from the control group, ^##^ *p* < 0.01 was significantly different from model group, △ *p* < 0.05 was significantly different from TGGR pair, ns > 0.05 was not significantly different.

During the whole experiment, the weight of mice in the control group steadily increased, while the weight of mice in the CUMS model group decreased significantly. After treatment with TGGR, the situation improved significantly, and there was no significant change in body weight after administration of 5 mg/kg EX-527 (Figure 5A). Secondly, the antidepressant effect of TGGR was evaluated by four behavioral manifestations (SPT, FST, TST, MWM). After 3 weeks of modeling, the sucrose preference score of each group of mice was significantly lower than that of the control group. After 7 weeks of modeling (4 weeks of simultaneous administration), the sucrose preference score of CUMS model mice continued to decrease, and the sucrose preference score of TGGR mice increased significantly (see Figure 5B). After EX-527 was applied, there was no significant effect on the sucrose preference score of mice. As shown in Figure 5C,D, the resting time for FST and TST of CUMS mice was significantly longer than that of the control group, indicating that the animals had anhedonia, decreased motor and exploration ability, and showed the core symptoms of depression. After the administration of TGGR, the resting time of mice was significantly shortened, and after the administration of EX-527, the resting time of mice was slightly extended. From the MWM tests in Figure 5E,F, it can be seen that the model mice spent less time in the target quadrant compared to the control group. These behavioral changes can be improved after TGGR treatment. The resting time of the mice after the administration of EX-527 did not change significantly. These results suggest that inhibition of the SIRT1-PGC-1α pathway does not significantly affect the improvement of depression-like behavior in CUMS mice.

### 2.8. Effects of TGGR on Depression-Related Indicators in CUMS Model Mice in the Presence of the SIRT1-Specific Inhibitor EX-527

ELISA was used to detect the plasma levels of 5-HT and MDA. Compared with the control group, the serum levels of 5-HT in CUMS model mice were significantly reduced. The concentration of 5-HT increased dramatically after the administration of TGGR, while the level of 5-HT did not change substantially after the administration of EX-527 (see Figure 5G). As shown in Figure 5H, compared with the control group, the serum MDA level of CUMS model mice was significantly increased after TGGR administration, while the serum MDA concentration of mice in the TGGR group was significantly decreased compared with the model group after TGGR administration. Compared with the TGGR group, 5-HT levels were slightly increased after administration of EX-527. These results suggest that inhibition of the SIRT1–PGC-1α pathway does not significantly affect the improvement of depression-related indicators in CUMS model mice.

### 2.9. Effects of TGGR on the Ultrastructure of Hippocampal Neurons in CUMS Mice in the Presence of the SIRT1-Specific Inhibitor EX-527

**Figure 6:**
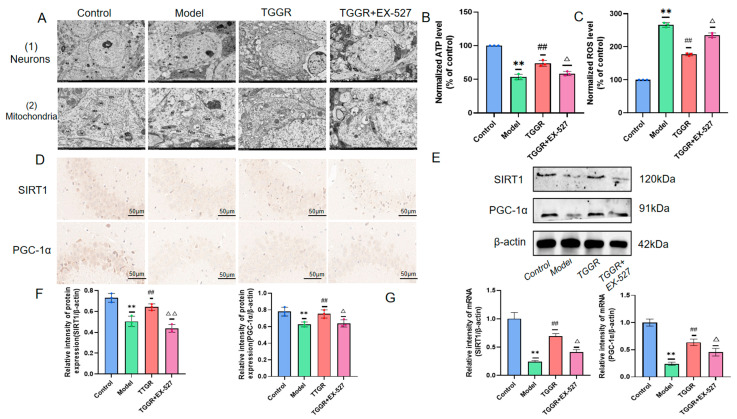
(**A**) Ultrastructure of hippocampal neurons and mitochondria of mice in each group. (**B**) ATP content in mouse hippocampus. (**C**) ROS levels in mouse brain tissue. (**D**) Expression of SIRT1 and PGC-1α in mouse hippocampus (immunohistochemical staining). (**E**) Western blot analysis of SIRT1 and PGC-1α in the hippocampus of mice in each group. (**F**) Protein expression normalized to β-actin for quantitative analysis, and its value expressed as an average. (**G**) SIRT1 and PGC-1α mRNA levels detected by RT-qPCR. Data are normalized to β-actin. Values are expressed as average ± SD. ** *p* < 0.01 was significantly different from the control group, ^##^ *p* < 0.01 was substantially different from model group, and ^△^ *p* < 0.05 and ^△△^ *p* < 0.01 were substantially different from TGGR group.

The SIRT1 inhibitor 5 mg/kg EX-527 was applied to mice, and the ultrastructure of hippocampal neurons was observed through transmission electron microscopy. Figure 6A(1) shows transmission electron microscopy (TEM) results. Compared with the control group, hippocampal neurons in the CUMS group displayed moderate edema, sparse cytoplasm, reduced electron density, clear membrane boundaries, local dissolution, and noticeable swelling of most organelles. The nucleus was elliptical, the nuclear membrane was intact, the perinuclear space was not widened, the chromatin distribution was not uniform, and the edge sets were occasionally seen. After administering 40 mg/kg TGGR, the neuronal cells displayed mild edema, the cytoplasm was uniform, the cell membrane was intact, and the organelles were partially swollen. The nucleus is elliptical, the nuclear membrane is intact, the perinuclear space is standard, and the chromatin is slightly agglutinated. After EX-527 was applied, the destructive effects of CUMS on the hippocampus resumed. These findings suggest RSV may affect nerve cell survival through the SIRT-1 pathway.

### 2.10. Effects of TGGR on the Ultrastructure and Function of Hippocampal Mitochondria in CUMS Mice in the Presence of the SIRT1-Specific Inhibitor EX-527

To investigate whether TGGR plays an antidepressant role by regulating the expression of SIRT1 and PGC-1α and improving the morphology and function of hippocampal mitochondria in mice, SIRT1 inhibitor Ex-527 was administered to mice, and the ultrastructure of hippocampal mitochondria was observed by transmission electron microscopy, as shown in Figure 6A(2). In the model group, most hippocampal mitochondria had increased swelling, a damaged membrane, a dissolved matrix, and a ridge. After TGGR administration, the degree of mitochondrial swelling was significantly reduced, the size was uniform, the membrane was relatively intact, the matrix was shallow, and the incidence of ridge breakage and shortening was reduced. After EX-527 was applied, the damaging effect of CUMS stimulation on the hippocampal mitochondrial structure was restored. Next, reactive oxygen species (ROS) levels in brain tissue were detected by 2, 7-dichlorofluorescein diacetate (DCFH-DA), and Adenosine triphosphate (ATP) levels in the hippocampus were detected by colorimetry. The quantitative results for ATP are shown in Figure 6B. Compared with the control group, the ATP level in the CUMS group was reduced, ATP consumption was significantly increased after TGGR administration, and the ATP level was again reduced after EX-527 administration. The quantitative results for ROS levels are shown in Figure 6C. Compared with mice in the control group, ROS levels in the hippocampal tissue of the CUMS mice were significantly increased.

In contrast, ROS formation in the hippocampal tissue of mice treated with TGGR decreased considerably. After the application of EX-527, ROS levels increased again. In summary, in the presence of SIRT1-specific inhibitors, the improvement effect of TGGR on the morphology and function of hippocampal mitochondria in mice is inhibited. These findings suggest that TGGR has a protective effect against mitochondrial damage induced by CUMS, and that this protective effect may depend on SIRT1 activity.

### 2.11. Effects of TGGR on the Expression of SIRT1 and PGC-1α Protein in the Hippocampus of CUMS Mice in the Presence of the SIRT1-Specific Inhibitor EX-527

Immunohistochemistry, Western blotting and RT-qPCR were used to detect the expression levels of SIRT1 and PGC-1α in the hippocampus of mice. The immunohistochemical results are shown in Figure 6D. After the administration of TGGR, the positive signal intensity of SIRT1 and PGC-1α in the model group was significantly increased compared with that in the model group, while the positive signal intensity of SIRT1 and PGC-1α was significantly decreased when the expression of SIRT1 was inhibited compared with that in the TGGR group. In addition, Western blot results in Figure 6E show that the expression levels of SIRT1 and PGC-1α were significantly reduced in the model group compared with the control group. However, compared with the model group, the expression levels of SIRT1 and PGC-1α were significantly increased after TGGR administration. After the administration of EX-527, the expression levels of SIRT1 and PGC-1α were again reduced compared to the TGGR group. The results of RT-qPCR in Figure 6F also show that the expression of SIRT1 and PGC-1α mRNA was significantly reduced in the model group compared with the control group. However, compared with the model group, the expression of SIRT1 and PGC-1α mRNA was significantly increased after TGGR administration. After EX-527 administration, the expression of SIRT1 and PGC-1α mRNA was again reduced compared to the TGGR group. These results indicate that TGGR regulates the expression level of PGC-1α through SIRT1.

## 3. Discussion

Depression is a common and severe mental illness that can cause significant psychological and physical damage [33]. The pathogenesis of depression is a complex multi-stage process involving multiple targets and multiple pathways [34,35]. Therefore, network pharmacology and multi-omics technology are often used to explore the pathogenesis of diseases and drug treatment methods [34]. Although network pharmacology has a good effect in exploring the synergistic effect of multi-components and multi-targets of traditional Chinese medicine, it also has certain limitations. For example, phytochemicals screened on the basis of oral bioavailability (OB) and drug similarity (DL) parameters may differ from those actually absorbed in the blood [36]. In addition, some of the major pathways produced by enrichment analysis may not have a clear association with the disease under study. Even if there is a relatively clear association with the disease under study, it is difficult to rule out whether it is dependent on the predicted target [36]. Moreover, the components of TGGR are diverse and specific. Therefore, differences in patient populations, disease severity, and reporting practices can greatly affect the accuracy and reliability of results. In addition, we recognize that transcriptomics also has certain limitations, such as the loss of spatial information due to cell dissociation during library preparation [37]. Therefore, more effort is needed to investigate these limitations and improve network pharmacology and transcriptomics.

Unfortunately, there are still few rapid, stable, and sustained antidepressants for clinical use [38]. As a natural product, TGGR has been shown to have potent therapeutic potential by modulating multiple biological mechanisms to improve depression-related symptoms, including neuroinflammation [39], immune disturbance [40], synaptic dysfunction, apoptosis [41,42], and so on. This study used a network pharmacology strategy combined with a whole-transcriptome sequencing approach to explore the mechanism of action of TGGR.

Consistent with previous studies, in this study, we observed depressive-like behavior in mice exposed to CUMS, and 40 mg/kg of TGGR effectively improved anxiety/depressive-like behavior and cognitive deficits in 7-week-old CUMS mice. At the same time, after TGGR administration, the irregular release of stress-related markers such as 5-HT and MDA in the serum of mice was significantly reversed, which helped to improve the depressed state of mice. In this study, the anxiety/depression-like behavior and cognitive impairment of CUMS mice were determined by SPT, FST, TST, and MWM. After TGGR treatment, the anxiety/depression-like behavior and cognitive impairment of CUMS mice were reduced.

After investigation and analysis, we found that the antidepressant effect of TGGR may be related to 71 potential targets of pharmacological networks and 845 transcriptomic DGEs. Combining network pharmacology and transcriptomic sequencing analysis identified eight key targets and 10 key signaling pathways. The key signaling pathways mainly involve the AMPK signaling pathway, PI3K/Akt signaling pathway, vascular endothelial growth factor (VEGF) signaling pathway, long-term depression, etc. Previous studies have found that the occurrence and treatment of depression are closely related to these pathways [43,44,45,46,47]. In the central nervous system (CNS), the PI3K/AKT pathway is a classical pathway that regulates neuronal survival and neurogenesis [48]. Protein expression of PI3K and AKT are significantly reduced in the brains of depressed mice, and fluoxetine can reverse this situation [49]. So far, there have been many studies on ginsenosides’ antidepressive effects through the PI3K/AKT signaling pathway, while there are few studies on ginsenosides’ effects on depression through the AMPK/SIRT1 signaling pathway, so we chose the AMPK/SIRT1 signaling pathway for follow-up experimental verification.

AMPK is a highly conserved serine/threonine protein kinase with anti-inflammatory functions that mediates catabolism and anabolism and changes the redox balance by regulating the expression or activation of its downstream molecules [38,50]. The AMPK signaling pathway has been identified to regulate depression-like behavior in animal models of CUMS [39].Moreover, SIRT 1 is a central downstream molecule in the AMPK signaling pathway and is closely associated with body energy metabolism processes, including neuronal damage and apoptosis [38,51]. Peroxisome proliferator-activated receptor-gamma coactivator 1-α (PGC-1α) is a major transcriptional coactivator of genes encoding proteins responsible for regulating mitochondrial biogenesis and function and is considered to be a downstream target of SIRT1 in a variety of biological effects, coordinating the regulation of genes required for mitochondrial biogenesis, which stimulates mitochondrial activity. The AMPK/SIRT1 pathway has been reported to have neuroprotective effects on neurons in neurological disorders such as Parkinson’s disease and Alzheimer’s disease [52]. At the same time, methods to improve activation of the AMPK and SIRT1 signaling pathways are also thought to enhance stress-induced depression-like behavior in animal models [53]. However, it is unclear whether ginseng can improve depression by adjusting the body’s energy metabolism through the AMPK/SIRT 1 signaling pathway.

Overall, activation of the AMPK/SIRT1 signaling pathway holds great promise as a strategy to facilitate depression-like behavior. Combining the results of network pharmacology and transcriptomic analysis, we found that the AMPK/SIRT1 signaling pathway mainly involves *LEPR*, *CD36*, *SLC2A4*, *SIRT1*, *PGC-1α*, and other genes. Transcriptomic analysis showed that *AMPK* in the model group was higher than that in the control group and TGGR group, while *SIRT1* and *PGC-1α* gene expression was lower than those in the control group and TGGR group. Therefore, we speculated that TGGR might alleviate depression by downregulating *AMPK* and upregulating the expression of *PGC-1α* and *SIRT1* genes. To further clarify whether the mechanism of TGGR regulating depression is related to the AMPK/SIRT1 signaling pathway, we used immunohistochemistry, immunoimprinting, and RT-qPCR to detect the protein expression level of this pathway. The results showed that TGGR increased the expression of SIRT1 and PGC-1α in the hippocampus of CUMS mice and decreased the expression of AMPK.

Meanwhile, PGC-1α is an essential downstream protein of SIRT1 because it regulates mitochondrial biogenesis and function. Mitochondria are the prominent organelles of cellular energy metabolism and reactive oxygen species (ROS) production [54]. In recent years, more and more studies have reported that the mitochondrial function and structure of patients with depression are abnormal, and the changes in mitochondrial ultrastructure can lead to energy metabolism disorders. Therefore, mitochondrial dysfunction is thought to be related to the pathogenesis of depression [55]. Discovering the importance of mitochondrial dysfunction in the pathogenesis of depression may help provide new therapeutic targets for the treatment of depression [56]. To verify whether TGGR can increase the expression of PGC-1α and affect the hippocampus mitochondria of mice, we observed and detected the morphology and function of hippocampus mitochondria by transmission electron microscopy and related mitochondrial function kits. In addition, to further verify whether changes in hippocampal mitochondrial morphology and function are caused by TGGR increasing SIRT1 and PGC-1α expression, we applied the SIRT1 inhibitor EX-527. The results showed that hippocampal neuronal injury could lead to mitochondrial morphological and functional disorders, a decrease in ATP levels, and an increase in mitochondrial ROS production. After TGGR administration, hippocampal mitochondrial morphology and function were improved in the CUMS group, while EX-527 restored the effects of CUMS on hippocampal mitochondrial morphology and function. Therefore, TGGR may play an antidepressant role by regulating the AMPK/SIRT1 signaling pathway and improving mitochondrial morphology and related functions in the hippocampus of mice.

## 4. Materials and Methods

### 4.1. Materials

TGGR (98%, yuanye BioTechnology, Shanghai, China); Selisistat (EX-527, an SIRT1 inhibitor, MedchemExpress, Shanghai, China); mouse 5-HT ELISA kit (Ruixin Biotechnology, Quanzhou, China); malondialdehyde (MDA) assay kit (TBA method) (Jiancheng Biotechnology, Nanjing,China); BCA protein assay kit, PMSF, RIPA lysis buffer, 5 × SDS-PAGE protein loading buffer (Servicebio Biotechnology, Wuhan, China); polyvinylidene fluoride (PVDF) membranes (0.45 μm, Millipore,Darmstadt, Germany); anti-AMPK (1:1000, Wanleibio, Shenyang, China); anti-SIRT1 (1:1000, Wanleibio, Shenyang, China); anti-PGC-1α (1:1000, Wanleibio, Shenyang, China), anti-β-actin (1:1000, Wanleibio, Shenyang, China); HRP goat anti-rabbit IgG (1:3000, Servicebio, Wuhan, China); ECL chemiluminescence kit (biosharp, Hefei, China); RNA keyTM reagent (Seven Biotechnology, Beijing, China); all-in-one first-strand cDNA Synthesis Kit II for qPCR (Seven Biotechnology, China); primers used for RT-qPCR (β-actin, AMPK, SIRT1, PGC-1α) (Servicebio Biotechnology, Wuhan, China); reactive oxygen species (ROS) test kit (012) (Beijing Biao Leibo Technology, Beijing, China); ATP content determination kit (Aidisheng Biotechnology, Yancheng, China)

### 4.2. Target Prediction and Disease Target Acquisition of Each Ginsenoside

Through literature review, several saponins with high ginseng root content were screened to obtain the ginsenosides Re, Rd, Rf, Rf, Rg3, Rc, Rg1, Rb1, and ginsenoside saponin diol [57,58,59,60]. We used the TCMSP (https://tcmsp-e.com/, Accessed on 2 April 2023) Swiss Target Prediction database (STP, http://www.swisstargetprediction.ch/, Accessed on 2 April 2023) 7 kinds of ginseng saponin database) and the chemical structure of the original ginseng diol molecules file. Through the PubChem and Drugbank (https://pubchem.ncbi.nlm.nih.gov/, Accessed on 3 April 2023) (https://go.drugbank.com/, Accessed on 3 April 2023) database for validation, and after verification, the obtained mol files were uploaded to the pharm-mapper (http://www.lilab-ecust.cn/pharmmapper/, Accessed on 4 April 2023) database to predict the targets of each ginsenoside. The predicted target names were normalized using the UniProt (https://www.uniprot.org/, Accessed on 4 April 2023) database. The protein Target components of depression were also derived from the TCMSP and Swiss Target Prediction database [25]. We used the UniProt database (https://www.uniprot.org/, Accessed on 5 April 2023) to change the name of the protein target to the corresponding gene symbol [25]. We searched the GeneCards database (https://www.genecards.org/ Accessed on 6 April 2023), online human Mendelian gene database (OMIM, https://omim.org/, Accessed on 6 April 2023), DrugBank database (https://go.drugbank.com/, Accessed on 7 April 2023), and therapeutic targets online database (TDD, http://db.idrblab.net/ttd/, Accessed on 8 April 2023) using keywords to identify the potential targets associated with ‘depression’ [25].

### 4.3. The Construction of the PPI Network

The acquired ginsenoside and the depression targets were uploaded to the Weishengxin (https://www.bioinformatics.com.cn/, Accessed on 9 April 2023) online platform for Venn mapping and obtaining the intersection genes. Then, the intersection genes were imported into the String (https://string-db.org/, Accessed on 10 April 2023) database for protein interaction analysis, and the analysis results were imported into Cytoscape 3.9.1 software. The plugin cytoNCA was used simultaneously for the analysis of results and the top 10 genes with degree value (degree) for visualization, as the experimental basis of target gene selection [61].

### 4.4. GO Functional Enrichment Analysis and KEGG Pathway Enrichment Analysis

The DAVID (https://david.ncifcrf.gov/, accessed on 15 April 2023) database was used for the intersection of genes for the Gene Ontology (GO) and Kyoto Encyclopedia of Genes and Genomes (KEGG) enrichment analysis, biological processes in KEGG enrichment analysis and GO analysis (Biological Process, BP), the molecular function (MF), and the cellular components (Cellular Component, the first 10 results of (CC)); these were then uploaded to the WeChat-credit platform for visual processing.

### 4.5. Construction of the “Drug-Target-Disease-Pathway” Network Map

According to the top 20 KGEE signaling pathways selected by the DAVID database and the intersection genes obtained in the above steps, the genes were sorted into network files and type files and imported into Cytoscape 3.9.1 software to build a “drug-component-target-pathway” network map.

### 4.6. Preparation of CUMS Mouse Model of Depression

The CUMS model was created with minor changes in the reference literature, including fasting, no water, day and night reversal, the 45-degree tilt of the cage, shaking of the cage, wet bedding, noise stimulation, ice water swimming, placing foreign objects in the cage, etc. [62]. The mice were subjected to random CUMS stimulation every day for 7 consecutive weeks, and their body weight was recorded weekly. After 3 weeks of modeling, the depression-like behavior of CUMS-induced mice was evaluated by sucrose preference test (SPT). Drug treatment began at week 4 after the initiation of the CUMS regimen and continued for 4 weeks. After the end of administration, the sucrose preference test (SPT), forced swimming test (FST), tail suspension test (TST), and Morris Water Maze (MWM) test were performed again. After the mice underwent behavioral tests, the mice were killed, and part of the hippocampal tissue was immersed in 4% paraformaldehyde. In contrast, other tissues were immediately stored in liquid nitrogen and preserved at −80 °C for future use.

### 4.7. Animals, Drugs, and Treatment

Male, 6-week-old, C57BL/6 mice (22–25 g) were obtained from Changchun Yisi Laboratory Animal Technology Co., LTD. (Changchun, China). The local institutional Animal care conducted all animal experiments and used guidelines approved by the Institutional Animal Care and Use Committee of Jilin Agricultural University. Animals were raised under standard conditions (23 ± 2 °C), with light and dark cycles for 12 h and free flow of food and water. The animals were fed for 1 week before the experiment. The mice were randomly divided into: control group, CUMS model group, CUMS + total ginseng saponins ginseng root (TGGR) (40 mg/kg/d) [55,63] group, and CUMS + TGGR + EX-527 (5 mg/kg/d) [64] group. TGGR (40 mg/kg, dissolved in saline) was administered via intragastric administration (ig) from week 4 to week 7. EX-527 (5 mg/kg, dissolved in saline) was injected intraperitoneally (ip) from week 6 to week 7. The control group was given normal saline.

### 4.8. Sucrose Preference Test

About 1–2 days before the SPT, sugar water (usually a 1% sucrose solution) was given to the animals to acclimate them to the taste of sugar water. On the day of the experiment, sugar water and regular water were put in two separate drinking bottles and labeled clearly. The drinking bottles of sugar and ordinary water were put into the animal cage simultaneously so the animals could freely choose to drink. We recorded the consumption of both liquids over a specific period (e.g., 12 h or 24 h). Liquid consumption could be measured by weighing or using a drinking bottle marked with a scale. We calculated the Sugar Water Preference Index (SPI): SPI = (sugar water intake/total intake) × 100%, where total intake is the sum of sugar water intake and regular water intake.

### 4.9. Forced Swimming Test

The mice were placed in a cylindrical bucket filled with 20 cm deep water at a temperature of 23 ± 2 °C for 6 min, and the resting state of the mice (when the mice floated or stopped struggling) was recorded for the last 4 min.

### 4.10. Tail Suspension Test

Mice were suspended 50 cm above the floor by adhesive tape placed approximately 1 cm from the tip of the tail. Each mouse was tested for 6 min, and the time during which the mouse remained immobile was recorded for the last 4 min [29].

### 4.11. Morris Water Maze

The water maze consisted of a circular water box with a 120 cm diameter and a platform complete with running water (22 ± 2 °C) placed 1–2 cm below the water surface [38]. The animals were put in one quadrant of the water maze and trained for five consecutive days to learn the platform position. With the camera installed above the water maze, the video tracking system (Smart 3.0, Panlab, Spain) was used to monitor the swimming trail of mice [38]. The time spent on test days to reach the platform was recorded. On day 6, the platform was removed, and the percentage of time mice spent in the target quadrant of the original platform was monitored [38]. The frequency of appearance in the target quadrant was analyzed.

### 4.12. Enzyme-Linked Immunosorbent Assay (ELISA)

Eyeball blood was centrifuged at 8000 rpm for 15 min at 4 °C, and the supernatant was collected. The 5-HT and MDA levels were measured using an ELISA kit according to the manufacturer’s instructions. The absorbance at the recommended wavelength for each well was measured using an ELISA meter (Epoch2; Burton Instruments Co., LTD, Gainesville, FL, USA).

### 4.13. RNA Extraction and Construction of RNA-Seq Libraries

Using Trizol reagent, total RNA was isolated from hippocampal tissue from mice in the Control, CUMS, and TGGR groups [CUMS + total ginseng saponins ginseng root (TGGR) (40 mg/kg/d)] (*n* = 3). Complementary DNA (cDNA) synthesis was performed after fragmentation of total RNA fragments into short fragments and enrichment of messenger RNA (mRNA) using oligo (dT) magnetic beads. The RNA was analyzed for integrity and purity using a NanoDrop 2000 (Thermo Fisher Scientific, Wilmington, DE, USA) to guarantee qualified samples for transcriptomics sequencing. After cluster generation, sequencing of the library preparations was performed using the Illumina HiSeq2500 platform provided by Bemec Biotechnology Co., Ltd. in Wuhan, China. Biomarker Technologies (Beijing, China) performed the entire RNA-Seq analysis. In fact format, the raw data underwent processing to remove reads containing adapters and those with low quality based on Q30 (an error rate of base calling ≤ 0.1%) to obtain clean and high-quality data.

### 4.14. Transcriptomic Analysis

The BMKCloud platform (https://international.biocloud.net/, Accessed on 6 December 2023) was used for dressing by screening DEG, adjusting the *p*-value by false discovery rate to *p* < 0.05 with the foldchanges ≥ 2 being set as statistically significant. Differential expression analysis is a method used to identify genes expressed differently between various samples or sample groups. Metascape (https://metascape.org/, accessed on 11 December 2023) was used for GO functional enrichment and KEGG pathway analysis, and Weishengxin (http://www.bioinformatics.com.cn/login/, accessed on 12 December 2023) was used for graphical visualization analysis.

### 4.15. Nissl Staining

Nissl staining was used to detect necrotic cell death according to morphological changes in the brain. Paraffin-embedded hippocampi were cut into 5 µm thick sections, and the sections were stained with 0.1% cresyl violet (Sigma Aldrich, Darmstadt, Germany), dehydrated with ethanol, and cover-slipped with Entellan. The staining was observed under a light microscope (400× magnification).

### 4.16. Immunohistochemical Staining

Immunohistochemical analysis of 5 µm thick paraffin-embedded sections of mouse hippocampus was performed. Then, they were incubated with SIRT1 (1:150) and PGC-1α (1:200) antibodies at room temperature for 4 h, shaken gently at 4 °C overnight, then washed with PBS 3 times and incubated with biotinylated secondary antibody at room temperature for 30 min. The sections were dyed with 3,3′-diaminobenzidine solution and then stained with hematoxylin. Five sections were selected from each animal, and the areas with SIRT1- and PGC-1α-positive cells were locally counted using Image J 2022 software.

### 4.17. Western Blot Analysis

We used Western blotting standard protocols to extract total protein from hippocampal tissue and then quantified the protein concentration with a bicinchoninic acid (BCA) protein assay kit. An equal amount of protein extract was loaded onto sodium dodecyl sulfate-polyacrylamide gel electrophoresis (SDS-PAGE) gel (80 V, 0.5 h, then 120 V, 1 h) and electrotransferred to a 0.45 µm polyvinylidene fluoride film. The isolated proteins were sealed with 5% skim milk at room temperature for 2 h and incubated with the primary antibody at 4 °C overnight. The next day, the second antibody was applied to the membrane at room temperature for 2 h, then washed with Tris-buffered saline and Tween 20 (TBST, 10 min each) at 3-round intervals. Finally, a Jena multifunctional imager (Analytikjena, Jena, Germany) was used to visualize the membrane, and the target protein for β-actin was standardized and quantified by Image J 2022 software.

### 4.18. Reverse Transcription-Quantitative Polymerase Chain Reaction

Total RNA was extracted from the hippocampus using the RNA keyTM reagent kit (Seven Biotechnology, Beijing, China), and the purity and concentration were measured by spectroscopy. The isolated RNA (1 μg) was reverse-transcribed into cDNA using a reverse transcription kit. The cDNA sample was added to the real-time PCR reaction solution and amplified by a real-time polymerase chain reaction (PCR) instrument. PCR primers were as follows:

SIRT1 upstream primer, ATCGTTACATATTCCACGGTGCT and downstream primer, CACTTTCATCTTCCAAGGGTTCT; PGC-1αupstream primer, GGATATACTTTACGCAGGTCGA, and downstream primer, CGTCTGAGTTGGTATCTAGGTC; β-actin upstream primer, GTCCCTCACCCTCCCAAAAG and downstream primer, GCTGCCTCAACACCTCAACCC.

The PCR amplification and uncoupling curves were confirmed after the reaction was completed. The relative expression levels of target genes were compared by two methods, ΔΔCt and β-actin mRNA.

### 4.19. Transmission Electron Microscopy

Hippocampal tissue (<1 mm^3^) was fixed with 2.5% glutaraldehyde for 2 h, then washed with BPS and fixed with 1% osmic acid for 2 h, and finally rinsed with PBS. Gradually, the tissue was dehydrated with different concentrations of ethanol and acetone, and then encased in acetone solution overnight. After fixation, the tissue was cut into 50 nm slices, stained with 3% uranium acetate and lead citrate, and then the mitochondrial morphology was observed under transmission electron microscopy.

### 4.20. Quantification of ATP

The content of ATP in the hippocampus was determined by colorimetry. The sample and the ATP standard were diluted in the ATP test lysate and mixed with the ATP test working solution according to the manufacturer’s instructions. The absorption value was detected using Biotek’s enzyme label, which adjusted the wavelength to 700 nm, and the ATP content in each sample was calculated according to the kit instructions. The results are shown as the percentage of controls.

### 4.21. Detection of Mitochondrial Reactive Oxygen Species

The fresh hippocampal tissue samples were washed with PBS. We accurately weighed 50 mg of tissue, added 1 mL homogenate buffer A, and thoroughly homogenized the mixture with a glass homogenizer. Next, the samples were centrifuged at 1000× *g*, 4 °C for 10 min, discard precipitation and take supernatant. Then, 190 μL homogenate supernatant and 10 μL 012 probes were added into a 96-well plate, blown with a pipette, mixed thoroughly, and incubated at 37 °C for 30 min without light. The fluorescence intensity was measured with an excitation wavelength of 488 nm and an emission wavelength of 530 nm.

### 4.22. Statistical Analysis

All data and statistical analysis complied with the recommendations on experimental design and analysis in pharmacology. Statistical comparisons were performed using one-way analysis of variance (ANOVA). GraphPad Prism 9.5.1 was used for all statistical analysis. *p* < 0.05 was considered statistically significant [29].

## 5. Conclusions

In summary, this study systematically explored the potential mechanism of TGGR in treating depression through network pharmacology, transcriptome sequencing analysis, and in vivo experiments. Our study confirms that TGGR exerts antidepressant effects through the AMPK-SIRT1 signaling pathway, and more importantly, we found that inhibiting SIRT1 expression in the mouse hippocampus can affect hippocampal mitochondrial morphology and related functions. Thus, this study demonstrates that strategies combining network pharmacology with transcriptomic analysis can help to explore and elucidate targets, pathways, and mechanisms of drug action in disease. This will provide a new theoretical basis for the research and application of TGGR in the field of depression treatment. It is emphasized that TGGR may be used to prevent people with chronic anxiety and high stress from becoming depressed, or may be used clinically for the early treatment of depression.

## Data Availability

The data that support the findings of this study are available from the corresponding author upon reasonable request.

## Supplementary Materials

The following supporting information can be downloaded at: https://www.mdpi.com/article/10.3390/ijms252312606/s1, Figure S1.
