# Supplementary figures and images for: Combining Network Pharmacology and Transcriptomic Strategies to Explore the Pharmacological Mechanism of Total Ginsenoside Ginseng Root and Its Impact on Antidepressant Effects
Source: Int J Mol Sci. 2024 Nov 24;25(23):12606. doi: 10.3390/ijms252312606 (PMC11640795; doi:10.3390/ijms252312606)

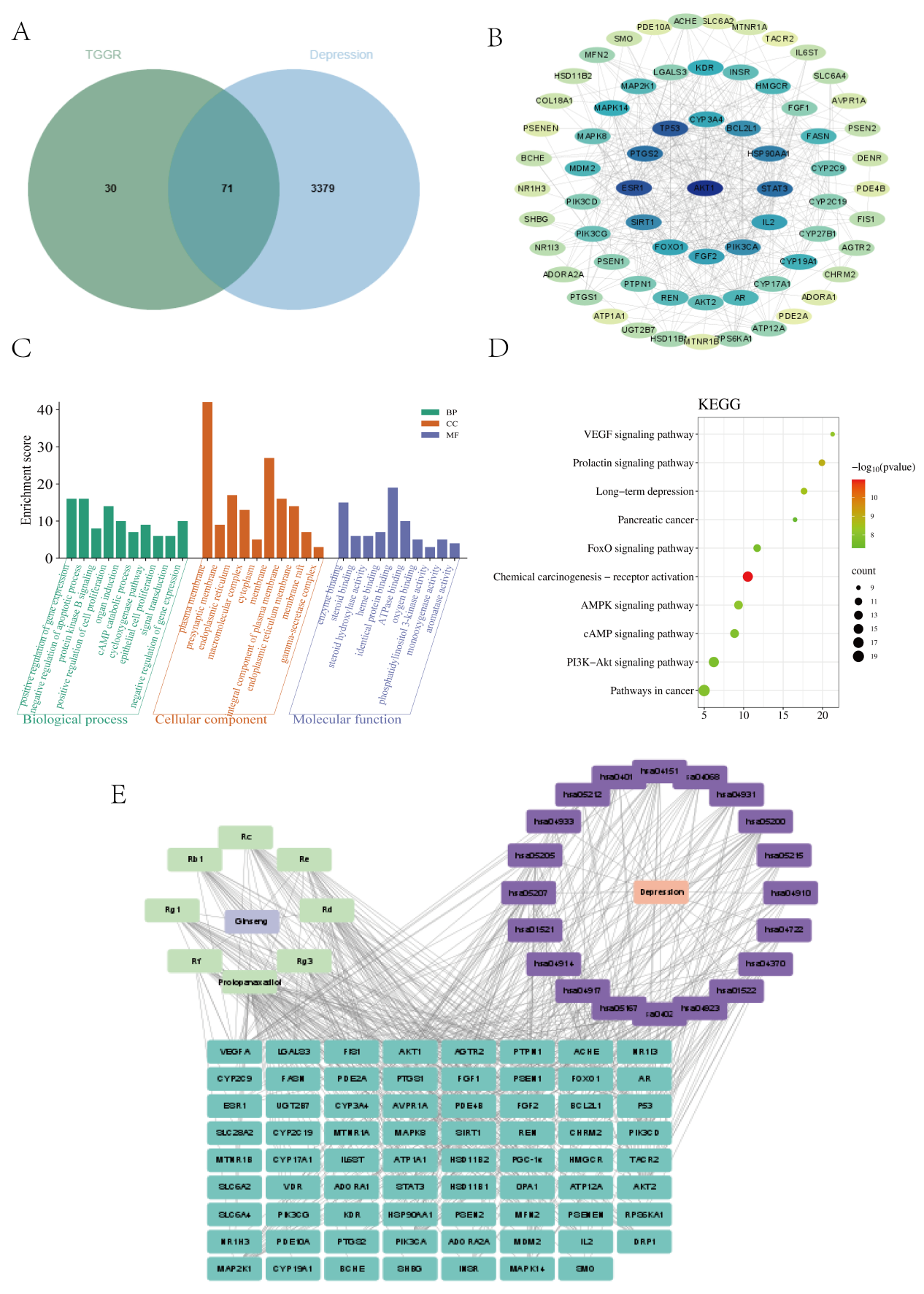

Supplement: Supplementary file 1 [file ijms-25-12606-s001.zip › ijms-3325795-supplementary.png]
